# Prevalence of suicidal ideation and suicide attempts in individuals with psychosis and bipolar disorder in South Asia: systematic review and meta-analysis

**DOI:** 10.1192/bjo.2023.570

**Published:** 2023-10-10

**Authors:** Ameer B. Khoso, Amna Noureen, Zaib Un Nisa, Alexander Hodkinson, Anam Elahi, Usman Arshad, Anum Naz, Mujeeb Masud Bhatti, Muqaddas Asif, Muhammad Omair Husain, Muhammad Ishrat Husain, Nasim Chaudhry, Nusrat Husain, Imran B. Chaudhry, Maria Panagioti

**Affiliations:** Division of At-Risk Mental State, Schizophrenia Spectrum, and other Psychotic Disorders, Pakistan Institute of Living and Learning, Karachi, Pakistan; and Division of Population Health, Health Services Research and Primary Care, School of Health Sciences, Faculty of Biology, Medicine and Health, University of Manchester, UK; Division of Child and Adolescent Mental Health, Pakistan Institute of Living and Learning, Karachi, Pakistan; Division of At-Risk Mental State, Schizophrenia Spectrum, and other Psychotic Disorders, Pakistan Institute of Living and Learning, Karachi, Pakistan; National Institute for Health and Care Research (NIHR) School for Primary Care Research, Division of Population Health, Health Services Research and Primary Care, School of Health Sciences, Faculty of Biology, Medicine and Health, Manchester Academic Health Science Centre, University of Manchester, UK; and National Institute for Health Research Greater Manchester Patient Safety Translational Research Centre, Division of Population Health, Health Services Research and Primary Care, University of Manchester, UK; Department of Primary Care and Mental Health, Institute of Population Health, University of Liverpool, UK; Department of Health Sciences, University of York, UK; Division of Substance-Related and Addiction Disorders, Pakistan Institute of Living and Learning, Lahore, Pakistan; and Division of Population Health, Health Services Research and Primary Care, School of Health Sciences, Faculty of Biology, Medicine and Health, University of Manchester, UK; Campbell Family Mental Health Research Institute, Centre for Addiction and Mental Health, Toronto, Canada; and Department of Psychiatry, University of Toronto, Canada; Division of Neurodevelopmental Disorders, Pakistan Institute of Living and Learning, Karachi, Pakistan; Division of Psychology and Mental Health, School of Health Sciences, Faculty of Biology, Medicine and Health, University of Manchester, UK; and Mersey Care NHS Foundation Trust, Prescot, UK; Division of At-Risk Mental State, Schizophrenia Spectrum, and other Psychotic Disorders, Pakistan Institute of Living and Learning, Karachi, Pakistan; Division of Psychology and Mental Health, School of Health Sciences, Faculty of Biology, Medicine and Health, University of Manchester, UK; and Department of Psychiatry, Ziauddin University, Karachi, Pakistan

**Keywords:** Suicidal ideation, psychosis, bipolar disorder, South Asia, suicide attempts

## Abstract

**Background:**

Suicidal ideation and attempts are growing public health concerns globally. Evidence from high-income countries suggests that individuals with psychosis and bipolar disorder are at increased risk of suicidal ideation and attempts, but there is a scarcity of evidence from South Asia.

**Aims:**

To estimate the prevalence of suicidal ideation and attempts in individuals with psychosis and bipolar disorder in South Asia.

**Method:**

In this systematic review and meta-analysis, four databases (PsycINFO, Web of Science, EMBASE and Medline) were searched until December 2022. Pooled prevalence was estimated with random-effects models. Heterogeneity was quantified with the *I*^2^-statistic.

**Results:**

The pooled sample size across the 21 studies was 3745 participants, 1941 (51.8%) of which were male. The pooled prevalence of suicide attempts in South Asian people with either psychosis or bipolar disorder was 22% (95% CI 17–27; *n* = 15). The pooled prevalence of suicidal ideation with psychosis or bipolar disorder combined was 38% (95% CI 27–51; *n* = 10). Meta-regression, subgroup and sensitivity analysis showed that the pooled prevalence estimates for both suicide attempt and ideation remained unaffected by variations in critical appraisal ratings and study designs. Only one study reported data on suicide-related deaths.

**Conclusions:**

One in four individuals diagnosed with psychosis or bipolar disorder have reported suicide attempts, whereas up to one in three have experienced suicidal ideation. These findings underscore the urgent need for clinicians to regularly assess and monitor suicidal ideation and attempts among individuals with these disorders in South Asia.

Suicide is a major public health concern globally, affecting not only the individuals but also family members and societies through emotional burden, financial costs and productivity loss.^1,2^ Suicidal behaviours (i.e. ideation and attempts) are major predictors of future suicide, and it is important to understand their prevalence to inform suicide prevention strategies. About three-quarters of global suicides occur in low- and middle-income countries (LMICs), which account for around 75% of the global estimated 800 000 annual deaths by suicide.^3^ In particular, South Asia represents a fourth of the global population, making it the most densely populated low-resource jurisdiction in the world. A recent scoping review including six South Asian countries reported higher suicide rates when compared with the average global suicides rates.^4^ Recently, nearly 0.25 million suicide deaths were reported in India, with suicide death rates 2.1 and 1.4 times higher in women and men, respectively, compared with the global average prevalence.^5,6^ Although rates of suicide attempts in other South Asian countries are lower compared with India – for instance, 3681 suicide attempts were reported in Sri Lanka and 22 675 were noted in Bangladesh – underreporting of suicide-related deaths and injuries, along with lack of available data and low-quality data, may contribute to underestimation of suicide rates in South Asia.^7–9^ In addition, a very recent systematic review reported a 5% pooled prevalence of suicide attempts among women, with young women being at increased risk of suicide attempts.^10^ This review concurs with the global burden study, which indicated an approximate 10% increment in suicide deaths in women compared with only about a 5% increase in men in the past three decades in India.^5,11^

## Suicide and mental illness

Suicidal behaviour and suicide can result from several risk factors, including genetic, biological, childhood, psychological, psychiatric and social influences.^12^ People with mental illnesses are especially found to be at increased risk of suicidal behaviour and suicide.^12,13^ Research mainly conducted in high-income countries shows that approximately 90% of people who die by suicide have a mental illness, with affective disorders accounting for 32–47% of cases, followed by schizophrenia (15–20%), personality disorders (8–11%) and alcohol dependence (8–17%).^14^ Psychosis including schizophrenia has a lifetime prevalence of approximately 3%, and bipolar disorder has a lifetime prevalence of approximately 2%,^15^ although these conditions are more common among people with lower socioeconomic status and educational level. Psychosis and bipolar disorder are considered chronic and disabling illnesses, which often worsen over the life course and are linked with higher disability, premature mortality, suicide and medical comorbidities worldwide.^1,16^

Systematic reviews consistently report that individuals diagnosed with psychosis^17,18^ and bipolar disorder^19,20^ are at increased risk for suicidal behaviour and suicide. Patients with schizophrenia who engage in suicide attempts and non-suicidal self-harm have a distinct clinical profile often showing early onset of symptoms, high rates of repeated suicidal behaviour and treatment delays.^21^ Likewise, bipolar disorder is associated with higher odds of suicide attempts compared with unipolar depression, and only a few promising pharmacological treatments have been identified to address suicide risk in people with bipolar disorder.^22,23^ A recent systematic review of observational studies reported that 33.9% of people with bipolar disorder had attempted suicide, with female gender positively associated with lifetime suicide attempts.^24^

Currently, there is a strong evidence that suicide, suicide attempts and suicidal ideation are prevalent in individuals with psychosis and bipolar disorder. However, this evidence is mainly derived from studies conducted in high-income countries. With over 40% of the poorest population, South Asia has approximately 150–200 million people experiencing psychiatric illnesses.^25,26^ Despite this, only limited research that has been conducted to understand the prevalence of suicide attempts, suicidal ideation and suicide in these individuals in South Asia. Moreover, no previous systematic review has synthesised the available evidence on the prevalence of suicide, suicide attempts and suicidal ideation in individuals with psychosis and bipolar disorder in South Asia. A recent systematic review synthesised suicide attempts and ideation in South Asia, but was limited to women and did not consider any clinical characteristics of participants.^10^

To address this major gap in the literature, this systematic review and meta-analysis aimed to examine the prevalence of suicidal ideation, suicide attempts and suicide-related deaths in individuals with psychosis and bipolar disorder in South Asia. The findings would be very helpful for guiding clinicians and policy makers in implementing suicide prevention strategies for high-risk groups in South Asia, such as people with psychosis and bipolar disorder.

## Method

The review protocol was registered on the International Prospective Register of Systematic Reviews (PROSPERO; registration number: CRD42019159106). We followed the guidelines of Preferred Reporting Items for Systematic Reviews and Meta-Analyses for reporting this review.^27^

### Search strategy and selection criteria

Initially, scoping searches were conducted to identify the relevant search terms. After that, four electronic databases (PsycINFO, Web of Science, EMBASE and Medline) were searched to identify published studies from inception until December 2022. The initial search was carried out in August 2020 and was subsequently updated until December 2022.

The following search terms were used: (‘suicid*’ OR ‘suicid* attempt’ OR ‘self-harm’ OR ‘deliberate self-harm’ OR ‘DSH’ OR ‘fatal self-harm’ OR ‘self-injur*’ OR ‘self-poison*’ OR ‘self-cutting’ OR ‘self-burn’ OR ‘self-mutilation’ OR ‘self-destruction’ OR ‘suicidal intent*’ OR ‘suicidal ideation’ OR ‘suicidal thought*’ OR ‘suicidality’) AND (‘psychosis’ OR ‘psychoses’ OR ‘schizo*’ OR ‘bipolar’ OR ‘affective’ OR ‘mania’ OR ‘severe mental illness*’ OR ‘serious mental illness*) AND (‘prevalence’ OR ‘epidemiology’ OR ‘rate’ OR ‘community based’ OR ‘population-based’ OR ‘cross-sectional’ OR ‘case-control’ OR ‘cohort studies’ OR ‘randomized controlled trial’ OR ‘RCT’). Subsequently, records were limited only to South Asian region with following search terms: (‘South Asia*’ or ‘India*’ or ‘Bangladesh*’ or ‘Pakistan*’ ‘Bhutan*’ or ‘Afghanistan*’ or ‘Maldive*’ or ‘Nepal*’ or ‘Srilanka*’). The reference lists of included studies were also searched to identify potential studies, and corresponding authors were contacted for additional papers in relevant domains (for the complete search history, see Supplementary Material available at https://doi.org/10.1192/bjo.2023.570).

### Definition of suicide-related terms

We defined suicide-related terms by referring to the glossary of terms from the United States National Suicide Prevention Strategy,^28^ whereas definition of psychosis and bipolar disorders were based on the recent version of the DSM-5-TR.

Suicide was defined as death from injury, poisoning or suffocation, where there is evidence that a self-inflicted act led to the person's death. Suicidal ideation was defined as self-reported thoughts of engaging in suicide-related behaviour. Suicide attempt was defined as an act where an individual engages in behaviour that has the potential to cause self-harm, but does not result in a death. However, this act underlies clear evidence that indicates that person intends to kill themselves, although injuries may or may not occur as a result.

### Eligibility criteria

We included studies that employed quantitative research designs (e.g. correlational, cross-sectional, prospective designs, longitudinal, case–control, cohort studies, randomised controlled trials), which explored the prevalence of suicidal ideation or attempt or suicide in individuals with psychosis and bipolar disorder. Psychosis, bipolar disorder, suicidal ideation and suicide attempts were identified by examining studies that used a complete or subsection of any standardised diagnostic or monitoring tool, semi- or unstructured clinical interview, case histories, autopsy interviews and/or clinical judgements made by a consultant psychiatrist or psychologist. Given the lack of available data in South Asia and to produce a comprehensive review that utilised all available evidence, no restrictions were placed on mental comorbidity associated with bipolar disorder and psychosis. The South Asian Association for Regional Cooperation includes Afghanistan, Bangladesh, Bhutan, India, the Maldives, Nepal, Pakistan and Sri Lanka. Studies based in countries other than these countries were excluded. We only included peer-reviewed articles written in the English language. We excluded qualitative studies, case studies, narrative reviews, commentary and discussion articles.

### Study selection

Four research team members (A.B.K., A. Noureen, Z.U.N. and A.E.) independently screened (in pairs) titles/abstracts, using predefined inclusion and exclusion criteria. Where discrepancies occurred, these were resolved by discussion and the involvement of a fifth reviewer (M.P.), who decided whether studies should be included. Abstracts that did not fulfil the inclusion criteria were excluded. Where it was uncertain if studies meet inclusion criteria, they were retained for the next stage of screening. The full articles were screened by the same team of reviewers and any discrepancies among reviewers were resolved by discussion with the wider team. For any identified conference abstracts, lead authors were contacted to check if they had eligible data.

### Data extraction

Initially extraction was conducted between March and August 2021; however, after updating the searches, extraction was further updated in January 2023. A standardised Microsoft Excel, version 365 for Windows spreadsheet was used for data extraction, which was piloted with the first three studies and then further adapted. The independent reviewers (A.B.K., A. Noureen, Z.U.N. and A.E.) extracted the data for each study and agreed on discrepancies. M.P. supervised the data extraction. Data extraction included study characteristics, sample characteristics, study design, methodology and outcomes. Extracted data included study details (author, year, study location), source (peer reviewed), study design information (type of design, number of groups, sampling method, sample population), participant characteristics (target sample, age, gender), measures used (measure of suicidal ideation and suicide attempts, measure of bipolar disorder, measure of psychosis) and prevalence (prevalence of suicide attempts in psychosis and bipolar disorder).

### Assessment of risk of bias

Risk of bias for studies included in the review was assessed with an adapted version of the Agency for Healthcare Research and Quality risk-of-bias tool.^29^ This tool has been used multiple times in reviews related to self-injurious behaviour and its risk factors.^30^ The tool assesses the risk of bias across multiple domains, including the representativeness and description of the cohort, the methods utilised to ascertain diagnoses and measure outcomes, and whether analyses were appropriate and consideration of confounding variables. The domains are rated using the terms yes, no, partial and unclear. Two research team members (A. Noureen and Z.U.N.) independently rated the studies and resolved any discrepancies by discussion with team (M.P. and A.B.K.). Ratings were used to identify the similar or common risk of bias and highlight areas of strength.

### Analysis

The primary outcome of the analysis was the prevalence of suicide attempts among individuals with psychosis and bipolar disorder in South Asia. Secondary outcomes included the prevalence of suicidal ideation and suicide-related deaths. All data were pooled with the *metaprop* function^31^ in Stata version 17 for Windows. Freeman–Tukey random-effects models were used in all meta-analyses. Random effects are generally more conservative and have better properties in the presence of heterogeneity.^32–34^ Given the expected heterogeneity in studies, random-effects models were utilised. Pooled prevalence was reported as percentages bound by their 95% confidence intervals. Heterogeneity was quantified with the *I*^2^-statistic and estimated 95% confidence interval (0–40%, might not be important heterogeneity; 30–60%, might present moderate heterogeneity; 50–90%, might present substantial heterogeneity; 75–100%, considerable heterogeneity). We conducted subgroup analyses for each condition (psychosis and bipolar disorder), timeframe of suicide attempts and suicidal ideation (lifetime and current), and countries (India and Pakistan). Meta-regressions were performed to examine whether the results were moderated by gender (proportion of females). Sensitivity analyses were conducted to account for variations in study designs (only cross-sectional studies were retained) and risk-of-bias ratings (only studies with low risk of bias were retained).^35^ We inspected the symmetry of the funnel plots and used Egger's test to examine for publication bias in analyses.^36,37^

## Results

**Fig. 1:**
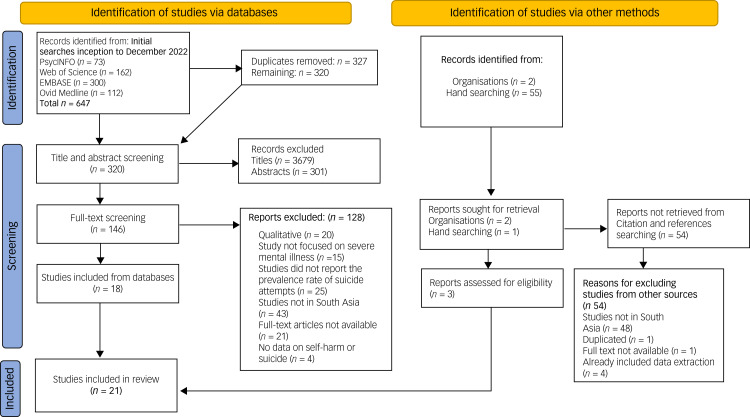
Preferred Reporting Items for Systematic Reviews and Meta-Analyses flow diagram.

Figure 1 presents the study selection process. A total of 704 records (647 from databases, 57 from other sources) were initially accessed. Following the removal of duplicates and the title/abstract and full-text screening, 21 studies met the inclusion criteria and were included in the review.

### Descriptive characteristics of included studies

The key descriptive characteristics of the included studies are presented in Table 1. The majority of studies took place in India (*k* = 18; 85%), with two in Pakistan (*k* = 2; 10%) and only one in Bangladesh (*k* = 1; 5%). All of the studies involved cross-sectional designs (*k* = 14; 67%), with the exception of four studies. These four studies involved retrospective longitudinal,^46^ case–control^56,62^ and prospective designs.^63^ Only one study was based in a community setting and 16 studies were based in hospital settings, including tertiary care mental health facilities and general hospitals. Three studies did not report information about the setting they were based in. The earliest study was published in 2000 and the most recent was published in 2022. The pooled sample size across the 21 studies comprised 3745 participants (psychosis *n* = 2096, bipolar disorder *n* = 1676), with a combined slightly higher percentage of males (*n* = 1941, 51.8%) than females (*n* = 1804, 48.2%); three studies did not report the gender composition of their sample. Validated psychiatric rating scales such as the Positive and Negative Syndrome Scale and the Mini-International Neuropsychiatric Interview (MINI) were used in 18 studies to diagnose psychosis and bipolar disorder. Three studies relied on clinical judgement by consultant psychiatrists and psychologists, and one study did not provide diagnostic information. For assessing suicidal ideation and attempts, nine studies used validated tools such as the Suicidality – Management, Assessment and Planning of Care and Beck Suicide Inventory. Six studies used subsections of tools like the MINI and Hamilton Rating Scale for Depression, whereas the remaining six employed informal methods like semi-structured or autopsy interviews to assess suicidal ideation and attempts. Fifteen studies (71%) reported data on suicidal attempts, ten (48%) studies reported data on suicidal ideation and only one study reported data on suicide-related deaths. This study reported that two out of 100 individuals with psychiatric morbidity (2%) with schizophrenia died by suicide. Across the 15 studies that reported suicide attempts, three studies reported recent suicide attempts and 12 reported lifetime suicide attempts. Across the ten studies that reported suicidal ideation, seven studies reported recent or current suicidal ideation and three studies reported lifetime suicidal ideation. Fifteen studies (71%) focused on individuals with psychosis, and six (29%) studies focused on people with bipolar disorder. Four studies took an inverse approach and examined the prevalence of psychosis and bipolar disorder in South Asian people who had attempted suicide or thought about suicide.

**Table 1:** Study characteristics and prevalence of suicidal ideation and attempts in psychosis and bipolar disorder

Author	Study design	Study settings	Sample population	Sample (*n*)	Age in years, mean (s.d.) [range] {median}	Measures for schizophrenia, bipolar disorder, suicide, suicidal ideation and suicide attempts	Prevalence of suicidal ideation/suicide attempt
Grover et al (2022) India^46^	Observational (retrospective longitudinal data)	Tertiary care hospitals	Bipolar disorder type 1, bipolar disorder type 2	*N* = 773 (mean 492, *F* = 281)	45.66 (±10.50) [17–75]	Young Mania Rating Scale Hamilton Rating Scale for Depression	Lifetime suicide attempts were observed among 242 (31%) patients with bipolar disorder
Nath et al (2021) India^47^	Observational (cross-sectional)	Tertiary care mental health facilities	Schizophrenia spectrum disorder	*N* = 140 (mean 76, *F* = 64)	31.17 (±8.5)	Positive and Negative Syndrome Scale International Suicide Prevention Trial Scale for Suicidal Thinking (ISST)	About 25.7% had previously attempted suicide and about 29.3% of patients with schizophrenia were experiencing suicidal ideation
Pillai et al (2021) India^48^	Observational (cross-sectional)	Tertiary care hospital	Bipolar disorder type 1 Bipolar disorder type 2	*N* = 200 (mean 110, *F* = 90)	*F* = 36.3 (11.6) mean 41.4 (14.4)	Mini-International Neuropsychiatric Interview Plus (version 5.0)	15 out of 200 patients with bipolar disorder had previously attempted suicide
Xue et al (2021) Pakistan ^49^	Observational (cross-sectional)	Out-patient psychiatric clinics	Bipolar disorder type 1 Bipolar disorder type 2	*N* = 266 (mean 189, *F* = 77)	[28–44] {35}	Hamilton Rating Scale for Depression, 17 items	67% indicated suicidality of any level, 51% indicated mild/moderate suicidality and 16% indicated severe suicidality
Husain et al (2021) Pakistan^50^	Observational (cross-sectional)	In-patient and out-patient psychiatric clinics	Schizophrenia spectrum disorder	*N* = 298 (mean 176, *F* = 122)	31.39 (±9.101)	Calgary Depression Scale for Schizophrenia Positive and Negative Syndrome Scale	18% of participants reported suicidal ideation
Subramanian et al (2020), India^51^	Observational (cross-sectional)	Out-patient psychiatric clinic	Bipolar disorder type 1	*N* = 102 (mean 49, *F* = 53)	35.72 (±10.02) [19–59]	Mini-International Neuropsychiatric Interview 5.0. Barratt Impulsivity Scale	36.3% had a history of lifetime suicide attempt
Arafat et al (2018), Bangladesh^52^	Observational (cross-sectional)	Tertiary teaching hospital	Schizophrenia	*N* = 121 (mean 50, *F* = 71)	25.56 (±10.01) [18–60]	Semi-structured questionnaire (further details not given)	Schizophrenia was found in 25% of the respondents, bipolar depression was found in 6.61% of respondents
Rajkumar (2018), India^53^	Observational (cross-sectional)	Out-patient psychiatric facility	Psychotic disorder	*N* = 29 (mean 10, *F* = 19)	31.14 (±10.68) [14–55]	Clinical interviews (further details not given)	55.17% experienced suicide-related ideations, 20.69% had a history of suicide attempts
Jakhar et al (2017), India ^54^	Observational (cross-sectional)	Out-patient psychiatric facility	Schizophrenia	*N* = 270	30.92 (±10.158)	Ram Manohar Lohia Risk Assessment Interview (RML-RAI) Diagnostic Interview for Genetic Studies – Hindi Version (DIGS)	22.59% were at risk of self-harm; 10% attempted suicide, out of which 60% were male
Shrivastava et al (2017), India^55^	Observational (cross-sectional)	Tertiary general psychiatric centre	Schizophrenia	*N* = 60 (mean 41, *F* = 19)	26.5 (±4.6) [18–39]	Scale for Impact of Suicidality – Management, Assessment and Planning of Care (SIS-MAP) Positive and Negative Syndrome Scale (PANSS) Hamilton Rating Scale for Depression	13 participants attempted suicide
Shrivastava et al (2016), India^56^	Observational (case–control)	General hospital	Early psychosis	*N* = 60 (mean 31, *F* = 29)	26.5 (4.6)	Scale for Impact of Suicidality –Management, Assessment and Planning of Care (SIS-MAP) Brief Psychiatric Rating Scale Hamilton Rating Scale for Depression	30 admitted with history of suicide attempt
Kattimani et al (2016), India^57^	Observational (cross-sectional)	Not reported	Bipolar disorder type 1	*N* = 150 (mean 72, *F* = 78)	37.8 (9.8)	Columbia Suicide Severity Rating Scale NIMH Life Chart Methodology Clinician Retrospective Chart	23.3% reported a previous suicide attempt and 37.3% reported suicidal ideation
Verma et al (2016), India ^58^	Not reported	Psychiatric department in hospital	Schizophrenia and schizoaffective disorder	*N* = 175 (mean 107, *F* = 68)	33.07 (7.67) [18–50]	Pierce Suicide Intent Scale Hindi version of Diagnostic Interview for Genetic Studies Beck Cognitive Scale	22% attempted suicide
Umamaheswari et al (2014), India^59^	Observational (cross-sectional)	Not reported	Bipolar depression	*N* = 130 (mean 86, *F* = 44)	43.04 (11.96), [18–60]	Beck Depression Inventory Mini-International Neuropsychiatric Interview Beck Hopelessness Scale Brief Psychiatric Rating Scale	Suicidal ideation *n* = 65, suicide attempts *n* = 10 (15.38%)
Gania et al (2016), India^60^	Not reported	Not reported	Bipolar affective disorder	*N* = 56 (mean 34, *F* = 22).	27.96 (7.44)	Mini-International Neuropsychiatric Interview	56 (32.18%) participants attempted suicide
Banwari et al (2013), India^61^	Observational (cross-sectional)	Out-patient psychiatric department	Schizophrenia	*N* = 50 (mean 29, *F* = 21)		Beck Suicide Intent Scale Mini-International Neuropsychiatric Interview	34% of participants attempted suicide once or more
Manoranjitham et al (2010), India^62^	Observational (case–control)	Community (village)	Psychiatric morbidity	*N* = 100 (schizophrenia: two patients)	42.24 (20.69)	Psychological autopsy interview Structured Clinical Interview for DSM-III	Prevalence of suicide was 2%
Shrivastava et al (2010), India^63^	Observational (prospective follow-up)	Psychiatric treatment centre	First-episode psychosis	*N* = 61 (mean 43, *F* = 18)	31.8 (7.6%)	Suicidality was rated by clinical interviews and medical records Positive and Negative Syndrome Scale	24% reported previous suicide attempts, 26% reported experience of a suicidal crisis and 32.8% reported suicidal ideation
Babu et al (2008), India^64^	Observational (cross-sectional)	In-patient psychiatric unit	Postpartum psychosis	*N* = 82	23.3 (3.44)	Comprehensive Psychopathology Rating Scale	38% reported suicidal ideation, 18% had a history of suicide attempts
Bhatia et al (2006), India^65^	Not reported	Tertiary care facility	Schizophrenia or schizoaffective disorder	*N* = 460 (mean 255, *F* = 205)	31.3	Diagnostic Interview for Genetic Studies	107 (23.3%) participants reported lifetime history of suicide attempts
Bhatia et al (2000), India^66^	Not reported	Tertiary care teaching hospital	Schizophrenia	*N* = 24	Not reported	Suicidal Intent Questionnaire	Eight participants had suicidal ideation, six had suicidal planning and six had a history of suicide attempt

Studies are presented in reverse chronological order.

### Risk-of-bias assessment

The detailed results of the risk-of-bias assessment are presented in the Supplementary Material and Table 2. Fifteen studies (71%) at least partially met the criteria of the risk-of-bias assessment, indicating low risk of bias. The most common methodological problems were related to the measurement of the outcome, missing data, blinding of researchers and control of confounders in analyses. All studies used appropriate method of analysis. Suicidal ideation and attempts were mostly determined with single self-report items or continuous subscale measures such as the Calgary Depression Scale for Schizophrenia.

**Table 2:** Overview of assessment of study methodological quality and risk of bias

Study number	Authors	Unbiased selection of the cohort	Adequate description of the cohort	Validated method for asserting clinical status of participant group	Validated method for assessing predictor or risk variables	Validated method for assessing outcome for criteria variable	Outcome assessment blind to diagnostic/ participant status	Missing data is minimal	Analysis control for confounding	Analysis method appropriate
1.	Grover et al (2022), India^46^	Yes	Yes	Yes	Yes	Yes	Yes	Yes	Yes	Yes
2.	Nath et al (2021), India^47^	Yes	Yes	Yes	Cannot tell	Yes	Cannot tell	Yes	Yes	Yes
3.	Pillai et al (2021), India^48^	Yes	Yes	Yes	Yes	Yes	Cannot tell	Yes	Yes	Yes
4.	Xue et al (2021), Pakistan^49^	Yes	Yes	Yes	Yes	Partial	Yes	Cannot tell	Yes	Yes
5.	Husain et al (2021), Pakistan^50^	Partial	Yes	Yes	Yes	Yes	Yes	Cannot tell	Yes	Yes
6.	Subramanian et al (2020), India^51^	Yes	Yes	Yes	Yes	Cannot tell	Yes	Yes	Cannot tell	Yes
7.	Arafat et al (2018), Bangladesh^52^	Yes	No	Partial	Partial	Partial	Yes	Cannot tell	Yes	Yes
8.	Rajkumar (2018), India^53^	Partial	Yes	Partial	No	Partial	No	Cannot tell	Cannot tell	Yes
9.	Jakhar et al (2017), India^54^	Partial	Yes	Yes	Yes	Yes	No	Cannot tell	Yes	Yes
10.	Shrivastava et al (2017), India^55^	No	Yes	Yes	Partial	Yes	No	Yes	Yes	Yes
11.	Shrivastava et al (2016), India^56^	Partial	Partial	Yes	Yes	Yes	No	Cannot tell	Yes	Yes
12.	Kattimani et al (2016), India^57^	Yes	Yes	Yes	Yes	Yes	No	Cannot tell	No	Yes
13.	Verma et al (2016), India^58^	Yes	Yes	Partial	Partial	Partial	Cannot tell	Cannot tell	Cannot tell	Yes
14.	Umamaheswari et al (2014), India^59^	Yes	Yes	Partial	Yes	Yes	Cannot tell	Yes	Cannot tell	Yes
15.	Gania et al (2016), India^60^	Yes	Yes	Partial	No	Partial	No	Yes	Yes-	Yes
16.	Banwari et al (2013), India^61^	Yes	Yes	Yes	Yes	Yes	No	No	Yes	Yes
17.	Manoranjitham et al (2010), India^62^	Yes	Yes	Partial	Partial	Partial	No	Yes	Yes	Yes
18.	Shrivastava et al (2010), India^63^	Yes	Partial	Yes	Yes	Cannot tell	No	Cannot tell	Cannot tell	Yes
19.	Babu et al (2008), India^64^	Yes	Yes	Yes	Yes	Yes	No	Yes	Yes	Yes
20.	Bhatia et al (2006), India^65^	Partial	Yes	Yes	Cannot tell	Cannot tell	Cannot tell	Cannot tell	Cannot tell	Yes
21.	Bhatia et al (2000), India^66^	Yes	Yes	Cannot tell	Cannot tell	Yes	Yes	Cannot tell	Cannot tell	Yes

Studies are presented in reverse chronological order.

### Prevalence of suicide attempts

The pooled prevalence of suicide attempts in South Asian people with either psychosis or bipolar disorder was 22% (95% CI 17–27, *n* = 15 studies). Overall, there were large variations across the studies, as indicated by substantial to considerable heterogeneity (*I*^2^ = 87.65%, *P* = 0.000).

**Fig. 2:**
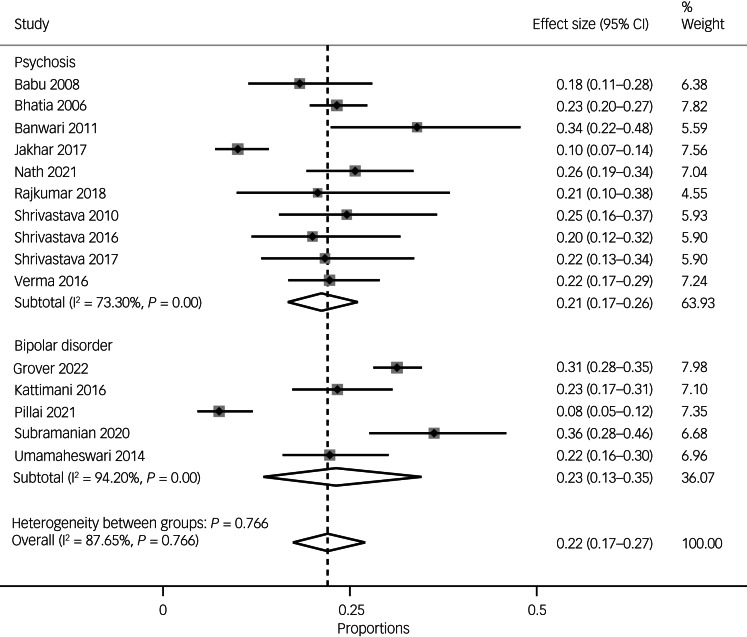
Pooled prevalence of suicide attempts overall and by diagnosis (i.e. psychoses versus bipolar disorder).

In terms of specific diagnoses, the pooled prevalence of suicide attempts in people with psychosis was 21% (95% CI 17–26, *I*^2^ = 73.30%, *n* = 10), and the pooled prevalence of suicide attempts in people with bipolar disorder was 23% (95% CI 13–35, *I*^2^ = 94.2%, *n* = 5) (see Fig. 2).

**Fig. 3:**
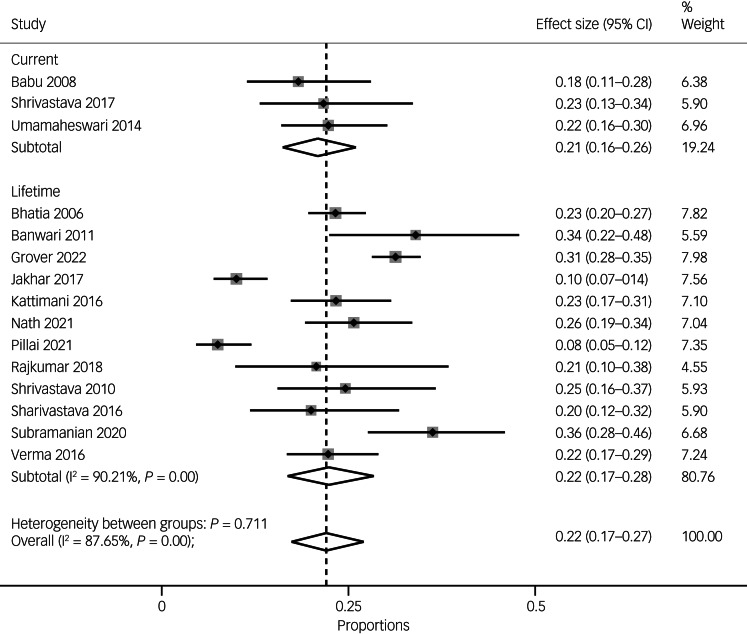
Pooled prevalence of suicide attempts overall and by duration (lifetime versus current).

The pooled prevalence of recent or current suicide attempts was 21% (95% CI 16–26, *I*^2^-statistic unavailable because of the small number of studies, *n* = 3), whereas the pooled prevalence of lifetime suicide attempts was 22% (95% CI 17–28, *I*^2^ = 90%, *n* = 10) (see Fig. 3).

We performed sensitivity analysis by retaining cross-sectional studies and studies with low risk of bias. We removed two retrospective^46^ and prospective^63^ studies, as well as all studies with high risk of bias. We found no significant differences in pooled prevalence (dropped from 21 to 19%, but confidence intervals for both estimates overlapped) and heterogeneity levels of suicide attempts (see Supplementary Fig. 1).

Meta-regression did not reveal a significant association between the proportion of females and the pooled prevalence of suicide attempts (see Supplementary Table 1).

Visual representation of the funnel plot indicated slight uneven distribution of study estimates against inverse standard error (see Supplementary Fig. 3), but Egger's test formally indicated the absence of any significant asymmetry in the funnel plot (beta1 = −0.03, *z* = −0.03, *P* = 0.97).

### Prevalence of suicidal ideation

The pooled prevalence of suicidal ideation for South Asian people with either psychosis or bipolar disorder was 38% (95% CI 27–51, *n* = 10), with considerable heterogeneity (*I*^2^ = 95.41%, *P* = 0.000).

**Fig. 4:**
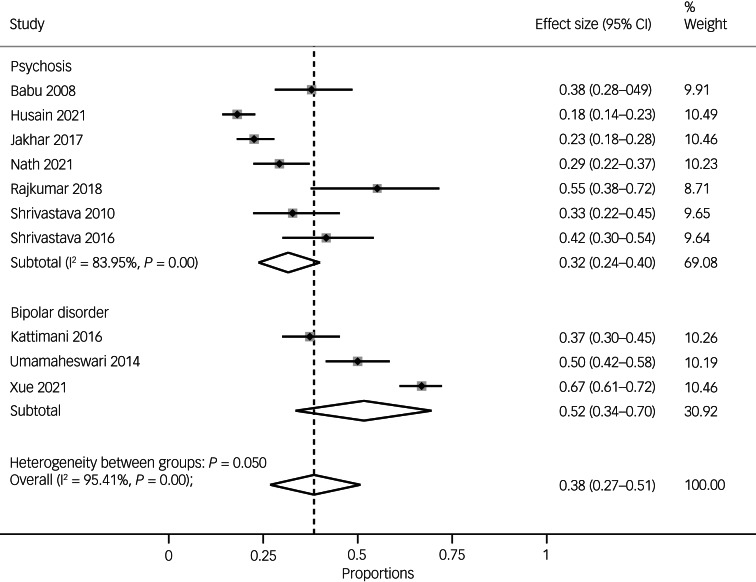
Pooled prevalence of suicidal ideation overall and by diagnosis (i.e. psychoses versus bipolar disorder).

In terms of specific diagnoses, the pooled prevalence of suicidal ideation in psychosis was 32% (95% CI 24–40, *I*^2^ = 83.95%, *n* = 7), and the pooled prevalence of suicidal ideation in bipolar disorder was 52% (95% CI 34–70, *I*^2^-statistic was unavailable because of the small number of studies, *n* = 3). There was considerable between-group heterogeneity (*P* = 0.05), indicating a higher rate of suicidal ideation in bipolar disorder compared with psychosis, but there were fewer studies in the bipolar disorder subgroup (see Fig. 4).

**Fig. 5:**
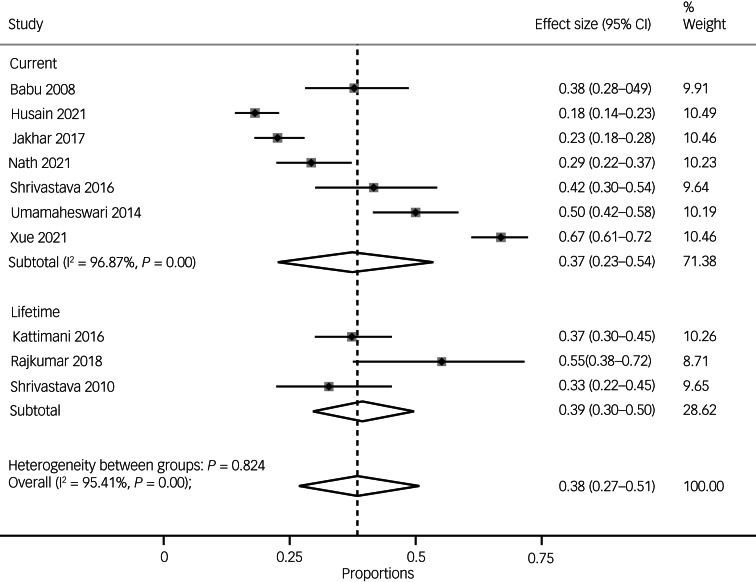
Pooled prevalence of suicide ideation overall and by duration (lifetime versus current).

The pooled prevalence of recent or current suicidal ideation was 37% (95% CI 23–54, *I*^2^ = 96.87%, *n* = 7), and the pooled prevalence of lifetime suicidal ideation was 39% (95% CI 30–50, *I*^2^-statistic was unavailable because of the small number of studies, *n* = 3) (see Fig. 5).

**Fig. 6:**
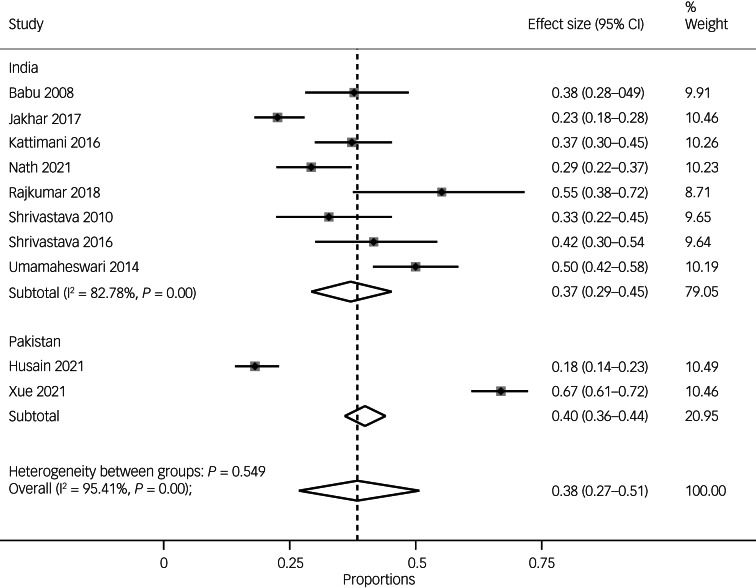
Pooled prevalence of suicide ideation overall and by countries.

The pooled prevalence of suicidal ideation in psychosis and bipolar disorder did not differ between India (37%, 95% CI 29–45%, *n* = 8) and Pakistan (40%, 95% CI 36–44%; I^2^-statistic unavailable because of the small number of studies, *n* = 2) (see Fig. 6).

Meta-regression did not show a significant association between the proportion of females and pooled prevalence of suicidal ideation (see Supplementary Table 2). After removing the studies with high risk of bias, the overall prevalence for suicidal ideation remained similar (see Supplementary Fig. 2).

The funnel plot showed that a few study estimates gathered on the top side of graph, indicating potential asymmetry (see Supplementary Fig. 4); however, Egger's test was non-significant (beta1 = 1.09, *z* = −0.65, *P* = 0.51).

## Discussion

### Summary of main findings

This is the first systematic review and meta-analysis examining the prevalence of suicide attempts, suicidal ideation and suicide among individuals living with psychosis and bipolar disorder in South Asia. The findings suggest that both suicide attempts and suicidal ideation are highly prevalent in South Asian individuals living with psychosis and bipolar disorder. Approximately one in four individuals with psychosis and bipolar disorder reported suicide attempts, and up to one in three individuals with psychosis and bipolar disorder reported suicidal ideation. Both suicidal ideation and attempts were more prevalent in people with bipolar disorder. Despite our comprehensive approach, we found scarce evidence about the prevalence of suicide-related deaths in people living with psychosis and bipolar disorder in South Asia.

### Comparison with previous literature

Our pooled prevalence estimates of suicidal attempts were nearly similar with the pooled estimates of previous meta-analyses of studies mainly based in high-income countries. These include recent meta-analyses that have shown that the prevalence of lifetime suicide attempts is 33.9% in people with bipolar disorder,^24^ 18% in people with first-episode psychosis and 11% in people with psychosis after 7 years of treatment.^38,39^ It is likely that the small sample sizes of most of the studies included in our analyses might have inflated our pooled prevalence estimates of suicide attempts. However, these high estimates may also reflect the concurrent presence of several risk factors for suicidal attempts among people with psychosis and bipolar disorder living in South Asian countries, including unemployment, low education level, low socioeconomic class, stigma toward mental illnesses and no reliable official data.^40,41^

### Implications for researchers, clinicians and policy makers

The review focused on the prevalence of suicide attempts, suicidal ideation and suicides among people with psychosis and bipolar disorder in South Asian countries, where suicide risk often remains under-addressed because of several common challenges, such as unemployment, disparities in health systems, poverty, the limited number of health professionals and limited investment in health by the government.^42^ The main implication of our findings is that suicidal ideation and suicide attempts among individuals with psychosis and bipolar disorder in South Asia are major, but overlooked, clinical and research concerns. Our pooled prevalence estimates of suicide attempts and suicidal ideation are high, but not definitive, because of the small number and low methodological quality of studies that have been conducted in South Asian countries. National epidemiological surveys and prospective studies are needed to accurately estimate the prevalence of suicides, suicide attempts (at present and lifetime) and suicidal ideation (at present and lifetime) in South Asia, and to identify subgroups of patients with psychosis and bipolar disorder that are at higher risk of suicide (e.g. based on health, demographic and social characteristics). Given the disproportionately higher burden of mental illness and suicide in LMICs, as well as the stigma associated with mental illness and suicide, it is imperative to address this global health concern by continuing to build on the sparse evidence base, to inform the development of cost-effective prevention strategies and policies in these settings.

In terms of clinical practice, the results highlight how important it is for clinicians to routinely assess and monitor suicidal ideation and suicide attempts in individuals with psychosis and bipolar disorder in South Asia. The lack of effective training of clinicians in suicide risk assessment, and the stigma and legal implications of suicide in South Asia, are key reasons why suicide attempts and suicidal ideation remain undetected and untreated. Many of the South Asian countries do not have effective policies, legislations, strategic plans, programmes and other instrumental measures to address mental health problems across communities.^40–42^ National policies and investments are therefore needed to develop culturally relevant, acceptable and effective suicide prevention strategies for individuals living with psychosis and bipolar disorder.

### Strengths and limitations

This is the first systematic review and meta-analysis to estimate the prevalence of suicidal ideation, suicide attempts and suicide in individuals with psychosis and bipolar disorder in South Asia. Given the dearth of literature on the topic, we combined lifetime and current suicide attempts in the analysis and included any observational study designs (although the majority of the studies were cross-sectional), to provide a comprehensive understanding of the overall prevalence of suicide attempts and suicidal ideation regardless of timeframe, and to make use of all available data. This review has several important limitations. The prevalence of suicidal attempts and suicidal ideation varied considerably across studies, as did the methods for their assessment. For example, variations in the timeframe of suicide attempts or suicidal ideation (current or lifetime) might be important when interpreting the differences in the prevalence estimates. A related concern is that across half of the studies (*n* = 9), it was unclear when did the suicidal attempt and suicidal behaviour occurred and whether it coincided or preceded the emergence of psychosis or bipolar disorder. Large longitudinal studies in community settings are needed to better understand the patterns of suicidal behaviours in people with psychosis and bipolar disorder; for example, to determine the timeframe and phase of illness in which they appear, and their duration and impact. Additionally, the measures used to assess suicidal ideation and attempts in the review were often single item and subscales taken from other instruments. Future research in South Asia would benefit from validated and dedicated measures of suicide attempts and suicide. The COVID-19 pandemic has greatly affected people with and without mental illnesses. Our updated searches did not find any eligible study on suicidal ideation and attempts among individuals with psychosis and bipolar disorder in South Asia during the COVID-19 pandemic. Finally, we do not know the severity and length of illness of participants with psychosis and bipolar disorder in the included studies. This could affect the prevalence estimates of suicidal ideation and behaviours; for example, in first-episode psychosis, the risk of suicidal attempts and suicide is greater in the beginning of the disease course.^42^ Similarly, suicide attempts and suicide in bipolar disorder vary significantly between illness phases, with mixed and depressive phases having the highest risk.^43–45^ Future studies should also consider the severity and duration of illnesses, to better understand the phenomenon of suicidal ideation and behaviours.

## Supporting information

Khoso et al. supplementary material 1 Khoso et al. supplementary material

Khoso et al. supplementary material 2 Khoso et al. supplementary material

## Data Availability

The data that support the findings of this study are available from the corresponding author, A.B.K., on reasonable request.
