# Supplementary material for: Prevalence of suicidal ideation and suicide attempts in individuals with psychosis and bipolar disorder in South Asia: systematic review and meta-analysis
Source: BJPsych Open. 2023 Oct 10;9(6):e179. doi: 10.1192/bjo.2023.570 (PMC10594255; doi:10.1192/bjo.2023.570)
Supplement: Khoso et al. supplementary material 2 — Khoso et al. supplementary material [file S2056472423005707sup002.docx]

Supplementary Material

**Figure 1:** Sensitivity Analysis for Suicidal Attempts (By Removing Studies at High Risk of Bias and Studies with Designs other than Cross-sectional)

**Figure 2:** Sensitivity Analysis for Suicidal Ideation (By Removing Studies at High Risk of Bias)

Table 1: Association of Suicidal Attempts with Gender: Meta Regression

Random-effects meta-regression Number of observations = 13

Method: REML Residual heterogeneity:

tau2 = .000583

I2 (%) = 7.64

H2 = 1.08

R-squared (%) = 63.78

Wald chi2 (1) = 1.73

Prob > chi2 = 0.1890

| _meta_es | Coefficient | Standard Error | z | p | 95% conf. interval | |
| --- | --- | --- | --- | --- | --- | --- |
|  |  |  |  |  | LL | UL |
| Female | .0002932 | .0002232 | 1.31 | 0.189 | -.0001443 | .0007307 |
| Constant | .2054297 | .0394009 | 5.21 | 0.000 | .1282054 | .2826541 |

Test of residual homogeneity: Q_res = chi2 (11) = 9.05 Prob > Q_res = 0.6174

Table 2: Association of Suicidal Ideation with Gender: Meta Regression

Random-effects meta-regression Number of observations = 8

Method: REML Residual heterogeneity:

tau2 = .0221

I2 (%) = 73.40

H2 = 3.76

R-squared (%) = 0.68

Wald chi2 (1) = 0.86

Prob > chi2 = 0.3542

| _meta_es | Coefficient | Standard Error | z | p | 95% conf. interval | |
| --- | --- | --- | --- | --- | --- | --- |
|  |  |  |  |  | LL | UL |
| Female | -.0017459 | .0018844 | -0.93 | 0.354 | -.0054392 | .0019474 |
| Constant | .5169141 | .1340797 | 3.86 | 0.000 | .2541228 | .7797054 |

Test of residual homogeneity: Q_res = chi2 (6) = 29.14 Prob > Q_res = 0.0001

**Figure 3:** Funnel Plot for Suicide Attempts

**Figure 4:** Funnel Plot for Suicidal Ideation

**Search History**

Database: APA PsycInfo <1806 to December Week 4 2022>

Search Strategy:

--------------------------------------------------------------------------------

1 (suicid* or suicid* attempt or self-harm or deliberate self-harm or DSH or fatal self-harm or self-injur* or self-poison* or self-cutting or self-burn or self-mutilation or self-destruction or suicidal intent* or suicidal ideation or suicidal thought* or suicidality).mp. [mp=title, abstract, heading word, table of contents, key concepts, original title, tests & measures, mesh word] (88729)

2 (psychosis or psychoses or schizo* or bipolar or affective or mania or severe mental illness* or serious mental illness*).mp. [mp=title, abstract, heading word, table of contents, key concepts, original title, tests & measures, mesh word] (336066)

3 (prevalence or epidemiology or rate or community based or population-based or cross-sectional or case-control or cohort studies or randomized controlled trial or RCT).mp. [mp=title, abstract, heading word, table of contents, key concepts, original title, tests & measures, mesh word] (594089)

4 (South Asia* or India* or Bangladesh* or Pakistan* or Bhutan* or Afghanistan* or Maldive* or Nepal* or Srilanka*).mp. [mp=title, abstract, heading word, table of contents, key concepts, original title, tests & measures, mesh word] (70012)

5 1 and 2 and 3 and 4 (73)

***************************

Database: Embase <1974 to 2022 December 30>

Search Strategy:

--------------------------------------------------------------------------------

1 (suicid* or suicid* attempt or self-harm or deliberate self-harm or DSH or fatal self-harm or self-injur* or self-poison* or self-cutting or self-burn or self-mutilation or self-destruction or suicidal intent* or suicidal ideation or suicidal thought* or suicidality).mp. [mp=title, abstract, heading word, drug trade name, original title, device manufacturer, drug manufacturer, device trade name, keyword heading word, floating subheading word, candidate term word] (166863)

2 (psychosis or psychoses or schizo* or bipolar or affective or mania or severe mental illness* or serious mental illness*).mp. [mp=title, abstract, heading word, drug trade name, original title, device manufacturer, drug manufacturer, device trade name, keyword heading word, floating subheading word, candidate term word] (508178)

3 (prevalence or epidemiology or rate or community based or population-based or cross-sectional or case-control or cohort studies or randomized controlled trial or RCT).mp. [mp=title, abstract, heading word, drug trade name, original title, device manufacturer, drug manufacturer, device trade name, keyword heading word, floating subheading word, candidate term word] (7542701)

4 (South Asia* or India* or Bangladesh* or Pakistan* or Bhutan* or Afghanistan* or Maldive* or Nepal* or Srilanka*).mp. [mp=title, abstract, heading word, drug trade name, original title, device manufacturer, drug manufacturer, device trade name, keyword heading word, floating subheading word, candidate term word] (561938)

5 1 and 2 and 3 and 4 (300)

***************************

Database: Ovid MEDLINE(R) and Epub Ahead of Print, In-Process, In-Data-Review & Other Non-Indexed Citations and Daily <1946 to December 30, 2022>

Search Strategy:

--------------------------------------------------------------------------------

1 (suicid* or suicid* attempt or self-harm or deliberate self-harm or DSH or fatal self-harm or self-injur* or self-poison* or self-cutting or self-burn or self-mutilation or self-destruction or suicidal intent* or suicidal ideation or suicidal thought* or suicidality).mp. [mp=title, book title, abstract, original title, name of substance word, subject heading word, floating sub-heading word, keyword heading word, organism supplementary concept word, protocol supplementary concept word, rare disease supplementary concept word, unique identifier, synonyms] (123255)

2 (psychosis or psychoses or schizo* or bipolar or affective or mania or severe mental illness* or serious mental illness*).mp. [mp=title, book title, abstract, original title, name of substance word, subject heading word, floating sub-heading word, keyword heading word, organism supplementary concept word, protocol supplementary concept word, rare disease supplementary concept word, unique identifier, synonyms] (362456)

3 (prevalence or epidemiology or rate or community based or population-based or cross-sectional or case-control or cohort studies or randomized controlled trial or RCT).mp. [mp=title, book title, abstract, original title, name of substance word, subject heading word, floating sub-heading word, keyword heading word, organism supplementary concept word, protocol supplementary concept word, rare disease supplementary concept word, unique identifier, synonyms] (6064926)

4 (South Asia* or India* or Bangladesh* or Pakistan* or Bhutan* or Afghanistan* or Maldive* or Nepal* or Srilanka*).mp. [mp=title, book title, abstract, original title, name of substance word, subject heading word, floating sub-heading word, keyword heading word, organism supplementary concept word, protocol supplementary concept word, rare disease supplementary concept word, unique identifier, synonyms] (400466)

5 1 and 2 and 3 and 4 (112)

***************************

Web of Science (Inception to December 2022)

(suicid* OR suicid* attempt OR self-harm OR deliberate self-harm OR DSH OR fatal self-harm OR self-injur* OR self-poison* OR self-cutting OR self-burn OR self-mutilation OR self-destruction OR suicidal intent* OR suicidal ideation OR suicidal thought* OR suicidality) AND (psychosis OR psychoses OR schizo* OR bipolar OR affective OR mania OR severe mental illness* OR serious mental illness*) AND (prevalence OR epidemiology OR rate OR community based OR population-based OR cross-sectional OR case-control OR cohort studies OR randomized controlled trial OR RCT) AND (South Asia* or India* or Bangladesh* or Pakistan* OR Bhutan* or Afghanistan* or Maldive* or Nepal* or Srilanka*) (162)
